# Caregivers’ and healthcare professionals’ perspective of barriers and facilitators to health service access for asthmatic children: a qualitative study

**DOI:** 10.1136/bmjresp-2021-001066

**Published:** 2021-12-22

**Authors:** Cristina Ardura-Garcia, John D Blakey, Philip J Cooper, Natalia Romero-Sandoval

**Affiliations:** 1 Institute of Social and Preventive Medicine, University of Bern, Bern, Switzerland; 2 Respiratory Medicine, Sir Charles Gairdner Hospital, Nedlands, Western Australia, Australia; 3 Medical School, Curtin University, Perth, Western Australia, Australia; 4 Escuela de Medicina, Universidad Internacional del Ecuador, Quito, Pichincha, Ecuador; 5 Institute of Infection and Immunity, St George’s University of London, London, UK; 6 Grups de Recerca d’Amèrica i Àfrica Llatines GRAAL Nodo Ecuador, Quito, Ecuador

**Keywords:** paediatric asthma, asthma in primary care

## Abstract

**Background:**

There is a high burden of asthma morbidity and mortality in Latin America. It has been proposed that this relates to limited access to diagnostic tests, asthma medications and specialised doctors. However, little is known of what caregivers of asthmatic children and healthcare professionals (HCPs) perceive as barriers and facilitators to adequate care. We aimed to explore the barriers and facilitators to asthma care access from caregivers’ and HCP’s perspective in an Ecuadorian low-resource setting.

**Methods:**

In 2017, we conducted 5 focus group discussions (FGD) with 20 caregivers of asthmatic children and 12 in-depth interviews with 3 paediatricians, 6 general doctors and 3 respiratory therapists in Esmeraldas city, Ecuador. FGDs and interviews were digitally recorded, transcribed, open-coded in QDA Miner, categorised using an interpretative phenomenological approach and analysed thematically. Barriers and facilitators were classified into availability, accessibility, acceptability and contact of healthcare services, based on Tanahashi model of health service access.

**Results:**

Limited resources, use of alternative medicines, fear of medication side-effects and lack of specific training for doctors and knowledge in families were common barriers for both caregivers and HCPs. Caregivers and HCPs proposed the implementation of public health asthma-focused programmes that would include close community-based follow-up of people with asthma, educational sessions for their families and public engagement activities. HCPs also suggested implementing training programmes on asthma management for general doctors.

**Conclusion:**

Multiple barriers identified by caregivers and HCPs referred to economic and health service organisational issues, fear of side effects of medication or ineffective self-management. Increasing caregivers and HCPs’ asthma knowledge, as well as HCPs’ communication skills to establish a patient-centred approach with a shared decision-making process could improve asthma care in this setting.

**Key messages**

- What are the barriers and facilitators to asthma health and home care access for children with asthma from caregivers’ and healthcare professionals’ perspective in an Ecuadorian low-resource setting?

- While some of the described barriers related to economic and health service organisational issues, others such as fear of side effects of medications or ineffective self-management could be overcome through educational interventions, both for caregivers and healthcare professionals.

- With this qualitative study, we have identified barriers and facilitators to availability, accessibility, acceptability and contact of healthcare services for children with asthma living in tropical America that may help improve asthma care in this setting.

## Introduction

Asthma is a chronic disease that requires a long-term collaborative approach to management, to ensure control of daily symptoms and avoid asthma attacks.^1^ Adequate health and home care access are essential to improve the management of children with asthma. However, inadequate asthma management is common, particularly in low-resource settings. In general, in Latin America, control of childhood asthma tends to be poor, resulting in preventable harm such as repeated use of emergency care and hospitalisations for asthma attacks.^2–5^ High asthma prevalence, lack of specialised doctors, poor access to asthma medications and lung function tests, lack of regular follow-up or low adherence to long-term medications are some of the factors considered to underlie the high asthma morbidity and mortality in Latin America.^6^

**Figure 1:**
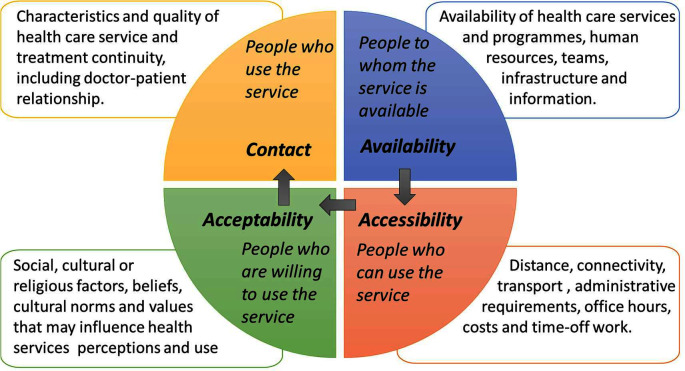
The four phases of Tanahashi’s model to obtain effective health service coverage.

Different models have been proposed to study access to health services. Tanahashi designed a model of effective health service coverage that includes factors related to health services and to the population distributed into four phases: availability, accessibility, acceptability and contact (figure 1).^7^ These phases have been used in a systematic review to classify reported barriers and facilitators to healthcare access for any disease,^8^ and could be applied also to asthma healthcare.

Several studies have investigated barriers and facilitators to health and home care access for children with asthma from the caregivers’ perspective,^9–25^ but only a few from the perspective of healthcare professionals’ (HCPs).^26 27^ Most such studies have been done in the USA and Europe. The few qualitative studies from Latin America^20–25^ have not examined the perspective of HCPs. Of particular importance is the examination of barriers and facilitators for children who have suffered an asthma attack, the population most at risk of long-term complications.^28 29^ In this study, we describe the barriers to and facilitators of asthma care, among children with a recent history of an asthma attack, from the perspectives of caregivers and HCPs in a low-resource setting in tropical coastal Ecuador.

## Methods

We did a qualitative study using a general inductive approach and an interpretative phenomenological analysis. This study was part of a cohort study that had recently followed-up children treated for an asthma attack at an emergency department (ED) in the city of Esmeraldas, Ecuador.^30^ We obtained informed written consent from all participants. We followed the Consolidated Criteria for Reporting Qualitative Research.^31^

### Setting

Ecuador is an upper-middle-income country, with a health system organised at three different levels. The first level covers health centres, some of which may include 24-hour emergency care, and are normally staffed by young HCPs. The second level covers general hospitals, where care is provided by general doctors and some specialists. The third level comprises specialised hospitals, situated in the major Ecuadorian cities, such as Quito and Guayaquil. Although access is universal, there are three different types of providers. The public health system is available for people (and their families) without regular employment. The second (the Ecuadorian Social Security Institute-Instituto Ecuatoriano del Seguro Social, IESS) is available for those with regular employment or who pay social security voluntarily. People treated at any of the public or IESS centres do not pay any contribution for the care received. Finally, private health centres and hospitals are also available to anyone able to pay for the services provided.

### Participants and recruitment

The study population was defined by purposive sampling, because we wanted to include participants who were knowledgeable about the phenomenon we aimed to study: asthma attacks in children.^32 33^ For this, we included HCPs actively involved in the acute management of asthmatic children in the city of Esmeraldas and caregivers of asthmatic children participating in the cohort study. We included HCPs from the two principal hospitals in the city (Hospital Delfina Torres de Concha, HDTC and the IESS Hospital) or primary care centre with emergency care. The HCPs included paediatricians, general doctors working at the ED and respiratory therapists. Nurses were not included, as they are not involved in the management of children with asthma in this setting. All were contacted by telephone. We used the snowball technique, by asking participating HCPs to identify additional colleagues who met inclusion criteria. For caregivers, we wanted to include caregivers of children treated for an asthma attack at an ED. As there are no electronic records and registries of these children in Esmeraldas, we contacted by telephone the families that participated in the cohort study and invited them to participate. Again, we used the snowball technique to encourage participating caregivers to inform other carers. None of the potential participants refused to participate, though 20 of the 40 caregivers that agreed to participate in the focus group discussions (FGDs) were not able to do so for practical scheduling reasons.

General doctors were less experienced and younger, paediatricians were older and more experienced, while respiratory therapists were between these groups in age and experience (online supplemental table 1). Most caregivers were mothers of children with asthma except for two grandmothers and two fathers. Most were homemakers (16/20) and of Afro-Ecuadorian ethnicity (13/20) (online supplemental table 2).

10.1136/bmjresp-2021-001066.supp1 Supplementary data

### Patient and public involvement

The families of children with asthma and the public were not directly involved in the design, recruitment or conduct of the study. Dissemination events about general concepts on asthma, its management and on the studies undertaken, were performed before and after the current study.

### Data collection

We performed in-depth semistructured interviews with HCPs and FGDs with caregivers between February and May 2017. Before interviews and FGDs, participants completed a short questionnaire to collect sociodemographic information.

We organised 12 interviews (three paediatricians, three respiratory therapists and six general doctors). We performed the interviews in a private room at the site of work of the HCP, at the study office or at the HCP’s home, depending on the interviewee’s preference and availability. Interviews lasted 35–55 min during which refreshments were offered. The interviews were undertaken by two of the authors: CA-G, a paediatrician who had worked previously at the ED of one of the hospitals, and NR-S, an experienced qualitative researcher with no previous contacts with HCPs in this setting. No additional paediatricians and respiratory therapist fulfilling our inclusion criteria were available in the city, and we stopped conducting interviews of general doctors once data saturation was achieved.

We organised five FGDs of 6–8 caregivers, although final numbers of participants were four in three FGDs, and five and three in the other two. Discussions took place in a spacious room in a health centre (housing the cohort study office) close to the main public hospital (HDTC). The discussions lasted about 1 hour, and refreshments were offered at the end. The FGDs were facilitated by CA-G (three FGD) and NR-S (two FGD). FGDs were stopped when data saturation was achieved. CA-G, the paediatrician in charge of the previous cohort study, had had sporadic contact with caregivers previously, and took all necessary precautions to avoid the influence of her previous contact, her knowledge of the healthcare system and asthma treatment, as well as to avoid any prior motivation. This was possible given the fact that the cohort study had finished the follow-up and CA-G did not have any recent direct contact with the families. Also, at the beginning of the FGD, CA-G clearly stated that these discussions would not affect the healthcare their children receive currently, and that they would only discuss the care received in the health centres and hospitals, and not during the cohort study. NR-S did not have any previous contact with the participating caregivers.

We designed a topic guide a priori based on previous field work and knowledge of the subject for interviews and FGDs. The topic guide included open ended questions and was modified as necessary after the first three interviews and the first FGD, using an emerging design (online supplemental material 1). All interviews and FGDs were digitally recorded and transcribed verbatim by professional transcriptionists. CA-G reviewed the transcriptions for comments and corrections.

### Data analysis

We used an inductive analysis and framework method to describe and examine the data. We used QDA Miner to support data management and analysis.^34^ CA-G individually coded each interview and FGD using an open coding process and descriptive codes. Each data segment could be given more than one code and classified into different categories. As part of the thematic framework, we used Tanahashi’s model’s domains, as identified codes fitted well into the model’s categories. Following development of the initial thematic framework, we reanalysed the data to assess appropriateness of fit, to allow modification and to highlight deviant cases. We included verbatim notes, field notes, a diary, the researcher’s reflective notes made during the research, and the transcribed recordings of the first three interviews and first FGD. Finally, we identified overarching themes that were common to the different Tanahashi’s categories. The analysis was supervised and guided by an experienced qualitative researcher (NR-S). The analysis was triangulated through meetings between CA-G and NR-S to discuss and agree on the coding system and thematic framework.

## Results

For each of Tanahashi’s domains, we here present the barriers and facilitators expressed by HCPs and caregivers. We provide an overview of the generated codes for each category in tables 1 and 2, and the overarching themes. We identified four main overarching themes: costs, logistic, adherence and education, with some overlap between the themes.

**Table 1:** Generated codes on barriers to health and home care access for children with asthma according to HCPs and caregivers, categorised into Tanahashi’s model elements

Category	HCPs	CGs
Availability	Lack of diagnostic tools, human resources and drugs (**LOGISTIC**) Lack of follow-up and asthma education programmes (**LOGISTIC and COST**) Lack of training for general doctors (**EDUCATION**) Mistrust in asthma drugs based on beliefs (**EDUCATION and ADHERENCE**)	Lack of medicines, medical supplies and diagnostic tests (**LOGISTIC**) Unclear, insufficient, contradicting and incorrect information from HCP (**EDUCATION**) Lack of training for general doctors (**EDUCATION**) Burden of care on women (**LOGISTIC**)
Accessibility	Cost of medicines, transport and specialist consultations (**COST**) Child cared by a third person (**LOGISTIC**) Health authorities not interested in asthma, not taken seriously CGs not given access to emergency department when child with asthma attack (**LOGISTIC**)	Economic and logistic difficulties (drugs, transport, days off-work) (**LOGISTIC & COST**) Difficulties in obtaining appointments (limited office hours and long waiting times) (**LOGISTIC**)
Acceptability	Use of natural remedies and other people’s advice by CGs (**EDUCATION**) Poor adherence to indications (**ADHERENCE**) Education and socioeconomic status of CGs (**EDUCATION**) Rejection of general doctors by CGs (**EDUCATION**)	Natural remedies and other people’s advice (**EDUCATION and ADHERENCE**) Reluctant to expose children to long-term medications (**EDUCATION and ADHERENCE**) Fear and mistrust towards doctors and hospitals (**EDUCATION and ADHERENCE**)
Contact	CGs do not listen or pay attention (**EDUCATION and ADHERENCE**) Lack of knowledge in CGs (not informed adequately) (**EDUCATION**) CGs take children only when severely ill (**EDUCATION**) Private HCW looking for profit (no prevention strategies) No sense of teamwork at ED (**LOGISTIC**)	Stop treatment if no symptoms (**ADHERENCE**) Ill-treated and abused by HCPs (**EDUCATION**) Not aware of effects, real side-effects and duration of the effect of asthma drugs (**EDUCATION**)

In capital letters and bold, we indicate the themes the codes were classified into.

CGs, caregivers; ED, emergency department; HCPs, healthcare professionals.

**Table 2:** Generated codes on facilitators and suggestions to improve health and home care access for children with asthma according to HCPs and caregivers, categorised into Tanahashi’s model elements

Category	HCPs	CGs
Availability	* Increase asthma knowledge for families and general population. (**EDUCATION**) * Organise an ‘Asthma Club’ for patients and families (**EDUCATION and LOGISTICS**) *Improve general doctor’s knowledge (**EDUCATION**)	* Increase asthma knowledge for families (**EDUCATION**) Feel empowered to manage their child’s asthma (self-efficiency) (**EDUCATION**) Enhanced knowledge improves child’s management (**EDUCATION**)
Accessibility	Free drugs and equipment available (**LOGISTICS and COSTS**) Possibility of families that can buy prescribed medicines or receive help (**COSTS**) * Periodic home visits, ‘Asthma Club’, public health asthma programmes (**LOGISTICS**)	Easy to get days off from school for children (**LOGISTICS**) * Organisation of a specialised area for asthma in the hospital or health centre (**LOGISTICS**)
Acceptability	CGs that do follow indications, treatment and control appointments (**ADHERENCE**) Taboos and myths easily eliminated with asthma education (**EDUCATION**) HCPs that care for the children and their families Greater implication of fathers in the city (**EDUCATION**)	Follow HCPs indications and treatment (**ADHERENCE**) Trust HCPs and drugs prescribed (over natural remedies) (**ADHERENCE**) Possibility of making older children responsible of their asthma management (**EDUCATION**) * Prefer a more holistic approach
Contact	CGs that cooperate, is easy to communicate with, and follow instructions (**EDUCATION**) Use of alternative methods to convey information (pictures, etc) (**EDUCATION**) Being honest and direct with CG (**EDUCATION**) HCPs motivated and happy when children improve or there is a feeling of teamwork	Adequate care by HCP, kind and quick (**EDUCATION**) Feel gratitude for guidance offered Importance of specific asthma programmes that increase awareness (**EDUCATION**)

In capital letters and bold, we indicate the themes the codes were classified into.

*Ideas or suggestions to improve health and home care access for children with asthma.

CGs, caregivers; HCPs, healthcare professionals.

### Availability

Most of the barriers identified in this category could be included in the overarching themes of logistic and education/training barriers. Caregivers and HCPs commented on the lack of diagnostic tools, trained doctors and medications for treating children with asthma. This lead to a mistrust in the diagnosis, a poor follow-up and inexistence of educational programmes for families of asthmatic children. Another barrier reported by paediatricians, was the lack of training programmes on asthma for general doctors. This was reflected in the way general doctors use their own experience instead of clinical guidelines to treat these children, in their mistrust of asthma medication’s side effects, and in the incomplete or misleading information that caregivers received regarding their child’s management. Caregivers stated that some HCPs had recommended them to take their children to another doctor, because they did not have the necessary training to manage their child’s asthma. Representative quotes are shown in table 3.

**Table 3:** Representative quotes for theme 1: availability and theme 2: accessibility

Availability	Barriers quotes	
	HCPs	‘There aren’t even enough human resources. It has happened to me sometimes that just one doctor oversees all the emergency area.’ [IG] ‘[I am used to diagnosing children with asthma] Clinically only. I don’t have, any methods, spirometry, nothing like that.’ [IP] ‘That’s what we are lacking, education programmes for asthmatic patients.’ [IT]
	CGs	‘But we took the control of the medicine ourselves, because they [at the public hospital] didn’t have anything. They didn’t even have disposable masks [to nebulize].’ [FGM] ‘The doctor tells you: ‘The child has this’. But never before he was checked up, he didn’t have any tests done, to say that this [asthma] is what the child really has.’ [FGG]
	Facilitators quotes	
	HCPs	‘It would be a matter of prevention, of communication, […], with speeches for people who are proactive in this management. To use the media, I don’t know, radio, television.’ [IP] ‘It is motivating for me that you are, doing this. Thank God, in the long term, well, I also continue with my studies, studying this [asthma] in more depth.’ [IG]
	CGs	‘If I know what the cause is, I would be more careful, making him not to be in contact with what hurts him, trying to keep him away. But if I don’t know, how am I supposed to know what I should protect him from?’ [FGM]
Accessibility	Barriers quotes	
	HCPs	‘Here in the city we don’t have a specialist in respiratory diseases, and in asthma and it is a little far.’ [IG] ‘They don’t give them authorization to be absent [from work] to have a medical check-up, just if the child is sick they get permission.’ [IP]
	CGs	‘To get an appointment sometimes you get an answer, sometimes you don’t and it is a chaos. One goes there, but they tell you: ‘Wait for your turn’. Then you stop giving him the medication.’ [FGM] ‘This moment he only has… I bought the blue one [salbutamol inhaler] for him. Because I didn’t have money for the other.’ [FGM]
	Facilitators quotes	
	HCPs	‘We give all the medication to them. We give them what the doctor prescribes. At least, for respiratory diseases, we provide them with all the medication. It is never missing’ [IT] ‘We could improve if we could visit mothers, be in touch with them by phone, […] creating an Asthma Club. […] we could […] visit his house, see the conditions, and have a complete dedicated team.’ [IP]
	CGs	‘Here at the hospital, there should be an area for that system only [asthma].’ [FGM]

CG, caregivers; FGG, focus group grandmother; FGM, focus group mother; HCPs, healthcare professionals; IG, interview general doctor; IP, interview paediatrician; IT, interview therapist.

HCPs and caregivers expressed ideas for improving availability of health and home care access for asthmatic children. For instance, the use of media, education campaigns in schools or an ‘Asthma Club’ for families, to increase asthma knowledge among caregivers and the general population. Some caregivers felt empowered to treat their child during an asthma attack. Experienced HCPs also discussed how asthma knowledge and management among general doctors might be improved through lectures and better in-job training. Some general doctors felt motivated to study more about asthma and allergies.

### Accessibility

The overarching themes identified in this category were costs and logistic barriers. A common complaint among HCPs and caregivers was the lack of specialised doctors to follow-up asthmatic children in this setting. They discussed the economic hardships and logistic difficulties experienced by the families to buy medicines not freely available, to access the health services, or to transfer the child to other specialists outside the city. The difficulties in arranging an appointment, the long waiting times at the emergency and the difficulties to get days off work, were the main barriers for healthcare accessibility for the caregivers, while the general doctors complained about the little time they had for each patient. HCPs explained how sometimes both parents had to work and leave the child under the care of third parties who might not manage the child’s asthma well. Finally, some HCPs and caregivers felt that asthma was not taken seriously by health authorities, resulting in a lack of preventive and follow-up programmes. Representative quotes are shown in table 3.

A facilitator that HCPs reported was the accessibility to free drugs and equipment at the hospital, and how some caregivers are able to buy the medication that may not be available at the hospital because of limited supply. HCPs proposed organising periodic home visits or a specific team to take care of children with asthma. Similarly, caregivers proposed assigning specific areas in the hospital for the management of these children. Also, caregivers commented that in general they found no difficulties in obtaining days off school for their asthmatic child.

### Acceptability

The main overarching themes identified in this category were adherence and education/training barriers. The caregiver’s beliefs and their use of traditional or natural remedies, was referred to by the HCPs and the caregivers as the main acceptability barriers to the asthma care offered by doctors. HCPs believed that this was caused by the families’ educational and socioeconomic level. On the other hand, caregivers described traditional medicine as part of their culture and beliefs, as a search for a solution not offered by ‘Western’ medicine, and as a consequence of their mistrust and fear towards hospital care. The reliance of caregivers on other people’s advice was also mentioned by HCPs and admitted by caregivers. These reasons, and the caregivers’ reluctance to long-term medication exposure for their children may explain the poor adherence to asthma treatments reported by HCPs. Another barrier reported by HCPs was that caregivers only take children to be treated during asthma attacks. General doctors felt that on some occasions they were disregarded by caregivers in their role as non-specialist doctors. Representative quotes are shown in table 4.

**Table 4:** Representative quotes for theme 3: acceptability and theme 4: contact

Acceptability	Barriers quotes	
	HCPs	‘First, the educational level of the parents […] I think it is crucial. Um, illiterate mothers or fathers can sometimes have the intention, but they don’t carry it out in a correct way. Second, the socio-economic level they belong to. […] they are the barriers to do it [follow the doctor’s instructions].’ [IG] ‘Here the idiosyncrasy makes people live from… from aromatic teas, from a certain plant’s tea, […] the witchdoctor who massages children. Then, a person here can’t adhere to medication.’ [IG]
	CGs	‘And do you know how it stopped [my child’s asthma]? Cockroach tea’ [FGM] ‘Because our body is not going to be under medication all the time; this medication has effects on certain parts of our organism and our defences. […] a lot of medication, […] makes them become stupid.’ [FGM]
	Facilitators quotes	
	HCPs	‘Education programs and health programs that teach what asthma is and that can remove all the taboos that we have about asthma.’ [IT] ‘Some of them do. Some come, even… and I give my cell number to those asthmatic patients. Some of them call me: ‘Doctor, my medicine is finished, help me with an appointment’.’ [IP]
	CGs	‘Because if the doctor tells me: ‘Give it to him for seven days and it has to finish all of it [the medication]’. I do.’ [FGM] ‘A ten-year old child, he can be aware. For instance, my daughter – she knows. Then when she tells me: ‘Oh, mom, I want a little [of something that may trigger her asthma]’. I say to her: ‘You decide if you take it, because you can take a little now, but your body will be affected’. Then […] she doesn’t take it.’ [FGM]
Contact	Barriers quotes	
	HCPs	‘[…] from the caregivers’ part there is total lack of knowledge too, eh…real lack of knowledge of the management, of the lifestyle that patients must have, in this case the caregiver ignores all this.’ [IT] ‘There are many parents who, when the attacks are over, they don’t go to follow-up consultations.’ [IG]
	CGs	‘Once I got there with – with my child with an attack and a nurse put a thermometer, I was there with the child, and she sat down, I swear that she took a nail polish, and she started to paint her nails.’ [FGM] ‘My child almost didn’t want to come, because he says that the other doctor is different. […] He said that the doctor is kind of angry.’ [FGM]
	Facilitators quotes	
	HCPs	‘I liked it because the mother was very cooperative, in this case. She understood me, I explained to her what we had to do, […] she started to give the medication to him properly.’ [IG] ‘I mean, making the parents aware, telling them the truth, being honest: ‘These are the conditions, and this is what may happen if you don’t do this’, and this kind of stuff.’ [IG]
	CGs	‘He was attended, he received first aid, but, no, he didn’t get better, but he was taken care of. I mean, I leave happy.’ [FGM] ‘We have to feel good for it, thankful because we have received advice, they have helped us […] It is for us to know how to cherish that. We have to learn from what the doctor offers us.’ [FGG]

CGs, caregivers; FGF, Focus group father; FGG, Focus group grandmother; FGM, Focus group mother; HCPs, Healthcare professionals; IG, interview general doctor; IP, interview paediatrician; IT, interview therapist.

When discussing facilitators for asthma care access, some HCPs believed that certain taboos and myths about asthma could be easily eliminated through education, and that caregivers’ cultural and educational level was sufficient to understand their indications. As such, they described how certain caregivers do follow their indications and attend regular follow-up appointments. Similarly, some caregivers expressed their trust in the doctors’ recommendations, even more than in traditional remedies, in some cases. However, some caregivers suggested the use of a more holistic approach to asthma care, that includes other recommendations or therapies apart from just medications. Finally, some caregivers commented on the possibility of making older children responsible for their own asthma care, and how this could improve the acceptability of the care provided.

### Contact or utilisation

Most caregivers described having been ill-treated or even abused by HCPs. They stated that a poor doctor–patient relationship might result in the child or their caregiver not wanting to attend that health service again. On the other hand, HCPs described that on occasions, they felt that caregivers did not seem to listen or understand them. HCPs believed that the child’s asthma care is the caregivers’ responsibility, and they blame them for the child’s poor asthma control, especially when they are not able to establish a continuous and close relationship with the caregivers. In contrast, some caregivers believed that the child’s poor asthma control is due to the poor asthma care received from the HCPs, and that this may be explained by the fact that their children were not followed up by the same HCP, who changed at every visit. Another barrier reported by some general doctors, was the lack of organisation and cooperation within HCP teams, especially in ERs. Representative quotes are shown in table 4. All the barriers mentioned in this category could be located under the overarching theme of education/training barriers, including the lack of training in provider–patient relationship for HCPs.

As a contact facilitator, some HCPs felt that communicating with caregivers was not difficult, especially if they had a higher educational level. On occasions, they reported meeting caregivers who were very cooperative, understanding the indications given and following them. Some caregivers reported having received adequate care, describing their satisfaction when treated promptly and with respect, even if the child did not improve as much as they expected. Some HCPs shared their methods to improve their communication with the caregivers, such as the use of drawings and games, or being honest and direct. Caregivers acknowledged this and expressed gratitude for the guidance received from some HCPs and for the fact that certain HCPs do follow-up their asthmatic children more closely. Some general doctors felt motivated and happy when caregivers turned up to inform them that their child was doing better. The same happened when HCPs in a department worked together as a team, cooperating and giving feedback to improve asthma management. Finally, some caregivers commented on the beneficial effect of a specific asthma programme (the cohort study during which they were followed up by phone every 2 months) on the home care of their children’s asthma.

## Discussion

### Main findings

In this qualitative study, we identified multiple barriers and some facilitators to availability, accessibility, acceptability and contact of healthcare services for children with asthma, as perceived by HCPs and caregivers. The barriers and facilitators could be summarised into four overarching themes: costs, logistic, education/training and adherence. Some barriers were common to both caregivers and HCPs, such as limited resources, the use of alternative medicines, the fear of side effects of asthma medications, and the lack of specific training for asthma management for doctors and asthma knowledge in families. Both caregivers and HCPs proposed the implementation of public health programmes focused on asthma including community-based follow-up of patients with asthma, and educational sessions for their families and public engagement activities. HCPs also suggested the use of specific training programmes for asthma management targeted at general doctors.

### Strengths and limitations

The main strength of this study was the inclusion of both HCPs and caregivers of asthmatic children, to contrast their opinions, beliefs and experiences. Similarly, the involvement of specialised doctors, respiratory therapists and young general doctors added value and richness to the discourses collected. This study has also some limitations. First, men (fathers) were underrepresented in the caregivers’ group discussions. Mothers and grandmothers are often charged with the child’s asthma care and were more likely to respond to the invitation to participate. This reflects the reality of home asthma care in this setting, although we were able to document the experiences of two fathers. Second, we were able to include relatively few experienced HCPs given there were no more paediatricians fulfilling our inclusion criteria. However, we did seem to reach data saturation in this subgroup of HCPs. Third, the study was undertaken in a low-resource urban setting, with a specific socio-cultural environment that may differ significantly from other settings, such as rural populations or high-resource settings. Nevertheless, our study site shares many characteristics with those of other similar settings in Latin America. Fourth, the level of education of the caregivers was higher than that of the background population. Finally, when studying access to healthcare, it would have been interesting to have included caregivers of children who did not choose or were unable to access healthcare services, as they may have encountered different barriers. We tried to include such families by using the snow-balling technique, but only families that had participated in the previous cohort study took part in the FGDs.

### Findings in relation to previous studies

There are few qualitative studies from Latin America or other low- and middle-income settings on barriers to healthcare access for children with asthma from caregivers’ and HCPs’ perspectives. In our study, we were able to contrast the opinions and experiences of the two sides, caregivers and HCPs, in contrast with most previous studies. This enabled us to understand the similar observations between the two sides, such as the lack of supplies and training in asthma care, as well as the divergent opinions, such as who is responsible for the child’s asthma care. Also, we focused on unscheduled healthcare for a child’s asthma attack, and its follow-up, while previous studies were focused mostly on chronic primary care. However, many of the barriers we identified were common to other settings. Both HCPs and caregivers in our study reported lack of asthma medications, medical supplies, human resources and diagnostic tools, as observed previously.^20 26^ The poor training of general doctors for adequate asthma management has been reported by general practitioners (GPs) in urban and rural areas in Australia,^26^ and this may be one of the causes for the scarce and contradictory information on asthma provided by HCPs to caregivers as described in this and previous studies.^12–15 18 19 24^ The costs and difficulties in obtaining days off-work for caregivers were also mentioned in this study by both HCPs and caregivers, and this issue has been raised frequently in previous studies.^10 13–15 17 20 21 26^ Caregivers’ beliefs, a mistrust in hospitals and doctors, combined with advice from non-HCPs, caused many to prefer the use of natural remedies over prescribed drugs. This was also the case for ethnic minority caregivers in the UK.^12^ The myths regarding side effects of asthma medications were very similar to those reported by caregivers in Peru and Colombia.^22 24^ This is a common barrier to asthma care, as for other chronic diseases, leading to poor adherence to medications.^12–15 17 18^ Poor adherence to long-term asthma medications and the fear of side effects is common in low,^22 24^ and high-income settings^9–15^ alike, and is also independent of ethnicity.^12^ In addition, HCPs also reported that caregivers used the health system only when acutely ill. The management of asthma as an acute illness (ie, during asthma attacks) rather than as a chronic disease has been reported frequently in Latin America^2–5^ including in a qualitative study from Brazil.^25^ Some of these barriers may be due to a poor provider-patient relationship. While HCPs felt ignored by caregivers, caregivers reported having been ill treated by HCPs in this study. Thus, HCPs felt that caregivers were responsible of the inadequate asthma care of the child, while caregivers believed that it was the HCPs’ responsibility. Lack of a shared decision-making process, discrimination, mistrust, and apportioning blame on the caregivers, are all aspects of the provider–patient contact that have been previously reported by caregivers of asthmatic children in other settings.^13–15 17^

Facilitators for health and home care access for children with asthma were not frequently reported in our study, though participants shared ideas for improvement. The main desire underlying these ideas was to improve knowledge and skills for asthma care among caregivers and general doctors. Caregivers in other settings,^13 16 18^ and GPs in Australia^26^ also expressed a need for additional training and information. Both HCPs and caregivers believed that the establishment of a specialised area within the hospital for asthmatic patients, public health asthma programmes and sanitary education on asthma would facilitate accessibility to healthcare and improve their management. Access to free asthma medications and to HCPs through an asthma control programme were described as facilitators to improve asthma control in children in Brazil.^23^ Also, some caregivers in our study expressed confidence in HCPs and the asthma treatment they prescribed. Trust in the medication prescribed was one of the facilitators identified in the systematic review by Hirmas *et al*^8^ and has been previously mentioned by caregivers of children with asthma.^12 24^ In addition, caregivers in other settings perceived their child’s asthma as well-managed and were satisfied with the care received,^12 13 15^ such as the mothers of children hospitalised for asthma in Peru, who spoke well of the nurses, both for their medical and human qualities.^21^ Similarly, some of the participant caregivers in our study reported having been treated kindly and adequately by HCPs. Once more, the inclusion of both caregivers and HCPs in the same setting using similar interview guides revealed how they both believe asthma care in children may be improved through specific programmes aimed at increasing asthma knowledge for families and HCPs, and providing closer follow-up.

### Implications for practice and future studies

Building a good patient–provider relationship is essential to improving asthma management. Being followed by the same professional or team who establish a patient-centred approach with a shared decision-making process, and which includes the child in these conversations, is vital for this.^13 15 19^ Training HCPs on shared decision making may help reduce the number of asthma attacks in children.^35^ Some of the identified barriers have already been extensively studied, such as the use of written asthma action plans or asthma educational interventions that have been shown to reduce the risk of asthma attacks.^36 37^ Others, such as home visits, the organisation of ‘Asthma Clubs’ for asthmatic patients and caregivers, or the use of media to increase the general public’s awareness on asthma are examples of interventions that could be studied further in this setting. Also, the involvement of specialised nurses in the management of these children and their families, and in the organisation of the ‘Asthma Clubs’ or home visits, should be further explored in this setting. Similarly, the establishment of a specialised centre for asthmatic patients with access to free medications and regular follow-up has been shown to reduce adult hospitalisations for asthma in Salvador, Brazil.^38^ A study of the effect on the patient’s asthma morbidity and quality of life, as well as the cost-effectiveness of such an intervention for asthmatic children in Esmeraldas would be extremely useful. All in all, both caregivers and HCPs in this setting agreed that more education and training for both caregivers and HCPs would improve asthma care by reducing the main barriers identified of costs, logistics, education and adherence.

Future studies should include people that may have been underrepresented here. For example, caregivers of children with asthma who do not use these health services, as perceived barriers may differ in this population. Also, male caregivers, although a minority among usual caregivers in this setting, may influence attitudes of female caregivers in the child’s house. Finally, the role of older children and adolescents should be studied because they may also influence home management of asthma and adherence to the treatment recommendations of HCPs.

## Conclusion

This study fills a knowledge gap by investigating barriers and facilitators around unscheduled healthcare and follow-up for children with asthma from the perspective of caregivers and HCPs. While some of the identified barriers related to economic and health service organisational issues, others such as fear of side effects of medications or ineffective self-management could be overcome through educational interventions, both for caregivers and HCPs. Increasing caregivers and HCPs’ asthma knowledge, as well as HCPs’ communication skills to establish a patient-centred approach with a shared decision-making process is likely to improve asthma care in this low-resource setting.

## Data Availability

Data are available on reasonable request.
