# Supplementary material for: Caregivers’ and healthcare professionals’ perspective of barriers and facilitators to health service access for asthmatic children: a qualitative study
Source: BMJ Open Respir Res. 2021 Dec 22;8(1):e001066. doi: 10.1136/bmjresp-2021-001066 (PMC8705230; doi:10.1136/bmjresp-2021-001066)
Supplement: Supplementary data [file bmjresp-2021-001066supp001.pdf]

# SUPPLEMENTARY DISPLAY ITEMS

## Caregivers' and health care professionals' perspective of barriers and facilitators to health service access for asthmatic children: a qualitative study.

Cristina Ardura-Garcia C[1], John Blakey[2,3], Philip J Cooper[4,5], Natalia Romero-Sandoval[4,6]

**Affiliations:**

1. Institute of Social and Preventive Medicine, University of Bern, Bern, Switzerland.
2. Respiratory Medicine, Sir Charles Gairdner Hospital, Perth, Australia
3. Curtin University Medical School, Perth, Australia
4. Escuela de Medicina, Universidad Internacional del Ecuador, Quito, Ecuador.
5. Institute of Infection and Immunity, St George's University of London, London, UK.
6. Grups de Recerca d'Amèrica i Àfrica Llatines GRAAL Nodo Ecuador, Ecuador

**Corresponding author:** Cristina Ardura-Garcia, Institute of Social and Preventive Medicine, University of Bern, Mittelstrasse 43, 3012 Bern, Switzerland. E-mail: crisardura@gmail.com



Ardura-Garcia C, *et al. BMJ Open Resp Res* 2021; 8:e001066. doi: 10.1136/bmjresp-2021-001066

**Supplementary Table 1:** Characteristics of health care professionals participating in the in-depth, semi-structured interviews

| Participant Code | Sex | Age | Ethnicity | Formal education | Years of experience |
|---|---|---|---|---|---|
| **IG1** | Female | 30 | Afro-Ecuadorian | General doctor | 3 |
| **IG2** | Female | 31 | Afro-Ecuadorian | General doctor | 5 |
| **IG3** | Male | 34 | Mestizo | General doctor | 6 |
| **IG4** | Male | 32 | Mestizo | General doctor | <1 |
| **IG5** | Female | 28 | Afro-Ecuadorian | General doctor | <1 |
| **IG6** | Female | 26 | Mestizo | General doctor | 1 |
| **IT1** | Male | 35 | Afro-Ecuadorian | Respiratory therapist | 8 |
| **IT2** | Female | 35 | Afro-Ecuadorian | Respiratory therapist | 8 |
| **IT3** | Female | 32 | Mestizo | Respiratory therapist | 6 |
| **IP1** | Female | 40 | Afro-Ecuadorian | Paediatrician | 12 |
| **IP2** | Male | 64 | Mestizo | Paediatrician | 27 |
| **IP3** | Male | 62 | Mestizo | Paediatrician | 26 |

*IG: In-depth interview general doctor; IT: in-depth interview respiratory therapist; IP: In-depth interview paediatrician.*

**Supplementary Table 2**: Characteristics of caregivers participating in focus group discussions

| Partici-pant Code | Sex | Age | Ethni-city | Educa-tion | Job | Relation to asthmatic child | Age asthmatic child[#] |
|---|---|---|---|---|---|---|---|
| **FG1-1** | Female | 32 | Af | Primary | House | Mother | 12/9 |
| **FG1-2** | Female | 25 | Af | Secondary | House | Mother | 7 |
| **FG1-3** | Female | 35 | Af | Secondary | Nursery | Mother | 9 |
| **FG1-4** | Female | 35 | Af | Primary | House | Mother | 12 |
| **FG2-1** | Female | 45 | Af | Primary | House | Mother | 8 |
| **FG2-2** | Male | 43 | Af | Secondary | Own business | Father | 8 |
| **FG2-3** | Female | 43 | Me | Primary | House | Mother | 7 |
| **FG2-4** | Male | 50 | Me | University | Secretary | Father | 9 |
| **FG3-1** | Female | 63 | Af | University | Retired* | Grandmother | 7 |
| **FG3-2** | Female | 37 | Me | University | Manager | Mother | 7 |
| **FG3-3** | Female | 31 | Af | University | House | Mother | 8 |
| **FG4-1** | Female | 60 | Af | Primary | House | Grandmother | 12 |
| **FG4-2** | Female | 32 | Af | Secondary | House | Mother | 7 |
| **FG4-3** | Female | 61 | Af | Primary | House | Grandmother | 12/10 |
| **FG4-4** | Female | 47 | Af | University | House | Mother | 14 |
| **FG4-5** | Female | 48 | Af | University | School teacher | Mother | 12 |
| **FG5-1** | Female | 30 | Me | University | House | Mother | 10 |
| **FG5-2** | Female | 45 | Af | Secondary | House | Mother | 11 |
| **FG5-3** | Female | 42 | Af | University | Nurse | Mother | 11 |
| **FG5-4** | Female | 33 | Af | Secondary | House | Mother | 11 |

**Used to work as an auxiliary nurse #: When two different ages appear, there are two different asthmatic children. FG: Focus group discussion, followed by the number of the group discussion – number of participant in that group; Af: Afro-Ecuadorian; Me: Mestizo.*



Ardura-Garcia C, *et al. BMJ Open Resp Res* 2021; 8:e001066. doi: 10.1136/bmjresp-2021-001066

## Supplementary Material: Interview guide

### 1. In-depth interviews

- What does the word 'asthma' mean to you?

- Please tell me about the last time you treated child with an asthma attack

- What do you think about the health and home care that asthmatic children receive?

- Please tell me about the last time you had contact with an asthmatic child's caregiver.

- What do you expect from an asthmatic child and his/her caregiver when you are treating them?

- Anything else you would like to say?

### 2. Focus group discussions

- What does the word 'asthma' mean to you?

- Please tell me about your child's last asthma attack

- What do you think about the health and home care that asthmatic children receive?

- Please tell me about the last time you had contact with a health care worker concerning your child's asthma.

- What do you expect from health care workers when you visit them for your child's asthma?

- Anything else you would like to say?



Ardura-Garcia C, *et al. BMJ Open Resp Res* 2021; 8:e001066. doi: 10.1136/bmjresp-2021-001066
